# Risk factors associated with tinnitus in 2948 Dutch survivors of childhood cancer: a Dutch LATER questionnaire study

**DOI:** 10.1093/noajnl/vdaa122

**Published:** 2020-09-15

**Authors:** Annelot J M Meijer, Marta F Fiocco, Geert O Janssens, Eva Clemens, Wim J E Tissing, Jacqueline J Loonen, Eline van Dulmen-den Broeder, Andrica C H de Vries, Dorine Bresters, Birgitta Versluys, Cécile M Ronckers, Leontien C M Kremer, Helena J van der Pal, Sebastian J C M M Neggers, Margriet van der Heiden-van der Loo, Robert J Stokroos, Alex E Hoetink, Martine van Grotel, Marry M van den Heuvel-Eibrink

**Affiliations:** 1 Princess Máxima Center for Pediatric Oncology, Utrecht, The Netherlands; 2 Department of Biomedical Data Science, Section Medical Statistics, Leiden University Medical Center, Leiden, The Netherlands; 3 Institute of Mathematics, Leiden University, Leiden, The Netherlands; 4 Department of Radiation Oncology, University Medical Center Utrecht, Utrecht, The Netherlands; 5 Department of Pediatric Oncology, Erasmus Medical Center – Sophia Children’s Hospital, Rotterdam, The Netherlands; 6 Department of Pediatric Oncology, University Medical Center Groningen – Beatrix Children’s Hospital, Groningen, The Netherlands; 7 Department of Hematology, Radboud University Medical Center, Nijmegen, The Netherlands; 8 Department of Pediatrics, VU University Medical Center, Amsterdam, The Netherlands; 9 Department of Pediatric Oncology, University Medical Center Utrecht – Wilhelmina Children’s Hospital, Utrecht, The Netherlands; 10 Department of Pediatric Oncology, Academic Medical Center – Emma Children’s Hospital, Amsterdam, The Netherlands; 11 Institute of Biostatistics and Registry Research, Brandenburg Medical School Theodor Fontane, Neuruppin, Germany; 12 Department of Medicine, Erasmus University Medical Center Rotterdam, Rotterdam, The Netherlands; 13 Dutch Childhood Oncology Group – Late Effects after Childhood Cancer (LATER) Registry, Utrecht, The Netherlands; 14 Department of Otorhinolaryngology, Head and Neck Surgery, University Medical Center Utrecht – Wilhelmina Children’s Hospital, Utrecht, The Netherlands

**Keywords:** childhood cancer survivors, hearing aid, late effects, ototoxicity, tinnitus

## Abstract

**Background:**

Tinnitus is a serious late effect of childhood cancer treatment. The aim of this study was to determine the occurrence and risk factors for tinnitus in a national cohort of childhood cancer survivors (CCS).

**Methods:**

Data were collected within the national Dutch Childhood Oncology Group - Long-Term Effects after Childhood Cancer (DCOG-LATER) cohort by a self-reported health questionnaire among 5327 Dutch CCS treated between 1963 and 2002. Siblings (*N* = 1663) were invited to complete the same questionnaire. Relevant patient characteristics and treatment factors were obtained from the Dutch LATER database. The occurrence of tinnitus in survivors was compared to siblings. To study the effect of risk factors, multivariate logistic regression models were estimated.

**Results:**

In total, 2948 CCS and 1055 siblings completed the tinnitus item. Tinnitus was reported in 9.5% of survivors and in 3.7% of siblings (odds ratio [OR] 3.0, 95% confidence interval [CI] 2.9–3.1). Risk factors associated with tinnitus in CCS were total cumulative dose cisplatin ≥400 mg/m^2^ (OR 2.4, 95% CI 1.4–4.0), age at diagnosis (≥10 years: OR 2.1, 95% CI 1.6–2.8), cranial irradiation/total body irradiation (TBI; OR 1.9, 95% CI 1.5–2.5), and neuro/ear, nose, throat (ENT) surgery (OR 1.8, 95% CI 1.1–2.9). Fifty-one percent of CCS with tinnitus had received treatment with either cisplatin, cranial irradiation/TBI, and/or neuro/ENT surgery.

**Conclusions:**

Tinnitus in CCS was present nearly 3 times more often than in siblings. Awareness in CCS previously treated with cisplatin, cranial irradiation/TBI, and/or neuro/ENT surgery is warranted. As only half of affected CCS had a history of these treatments, it seems that other factors might be associated with tinnitus occurrence in this population.

**Key Points**

- Childhood cancer survivors are at higher risk for tinnitus compared to siblings.
- Cisplatin, cranial irradiation, and CNS surgery are risk factors for tinnitus.
- Tinnitus screening after childhood cancer deserves serious attention.

**Importance of the Study**

Tinnitus is a serious late effect of childhood cancer treatment. Even though tinnitus can have a considerable impact on the quality of life, the disorder has not obtained major attention in survivorship care. This is disturbing, as tinnitus can result in sleeping difficulties, anxiety, depression, and even sporadically to self-harm and suicide. Studies on prevalence and risk factors of tinnitus after childhood cancer treatment are limited. The results of this study are important to increase awareness for tinnitus among clinicians and to identify groups of childhood cancer survivors at high risk of developing tinnitus. Our study may also facilitate the design of regular screening recommendations for the disorder along with audiological checkups in late effect clinics and in the long run lead to changes in treatment strategies to decrease tinnitus. Regular surveillance aids in (early) detection, referral, and treatment of tinnitus.

Over the past decades, outcome of childhood cancer has improved considerably.^1^ However, improvement in survival is associated with the development of late toxicities, among others due to increased treatment intensity. About 75% of childhood cancer survivors (CCS) suffer from one or more adverse events.^2,3^ Long-term morbidity after childhood cancer includes ototoxicity, which comprises damage to the cochlea resulting in permanent hearing loss, a condition often accompanied by tinnitus.^4^ Both hearing loss and tinnitus can have a severe negative impact on quality of life. Hearing loss impacts speech discrimination, resulting in decreased learning and social performance.^5^ Tinnitus can have an (additional) negative effect by causing stress, problems with coping, and diminished concentration.^6^

Compared to hearing loss, tinnitus is a less often investigated side effect after childhood cancer treatment. Patients with tinnitus suffer from ringing, buzzing, or hissing sounds in their ear or head that cannot be perceived by others. Its severity and accompanying symptoms vary between affected individuals.^7^ Currently, multiple hypotheses exist on mechanisms for tinnitus development. A recent understanding is that following cochlear or neural damage, neurons in the dorsal part of the cochlear nucleus (DCN) respond by upregulating both spontaneous and sound-evoked neural activity. This increase in spontaneous activity is caused by unbalanced excitatory and inhibitory nerve transmission, which leads to an increased firing rate of neurons and eventually to the perception of tinnitus. It is believed that damage to cochlear hair cells due to cisplatin may cause increased DCN activity.^8^

Audiological monitoring for hearing loss has become part of standard care during childhood cancer treatment, and recently international consensus was reached for hearing loss surveillance in CCS. In contrast, tinnitus as a late effect of childhood cancer treatment has not obtained major attention so far, and the lack of knowledge on prevalence and risk factors in survivors has been acknowledged.^9^ Therefore, in the current study, we studied the occurrence of tinnitus in a national cohort of CCS in comparison with healthy siblings and identified patient and treatment-related risk factors associated with the occurrence of tinnitus in CCS.

## Materials and Methods

### Study Population

Data for this cross-sectional study were collected within the Dutch Childhood Oncology Group - Long-Term Effects after Childhood Cancer (DCOG-LATER) study, a national study that has been set up to gain more insight into all long-term side effects of treatment among the earliest cohort of 6165 Dutch CCS. Included subjects were diagnosed with cancer before the age of 18 years, (co-)treated in 1 of the 7 pediatric oncology centers from 1963 to 2002, and alive (more than) 5 years after diagnosis. The characteristics of this cohort have been previously described.^10^

As part of this national study, a general health questionnaire was sent out between 2014 and 2016 to all eligible CSS alive in 2013. This list contained questions on current and past health status, family history, medication use, smoking, alcohol and drug habits, as well as physical activity. In addition, the questionnaire included queries on tinnitus and hearing aid use. Healthy siblings who were signed up by the survivors served as the control group for this study. They were asked to complete the same questionnaire.

**Figure 1.**
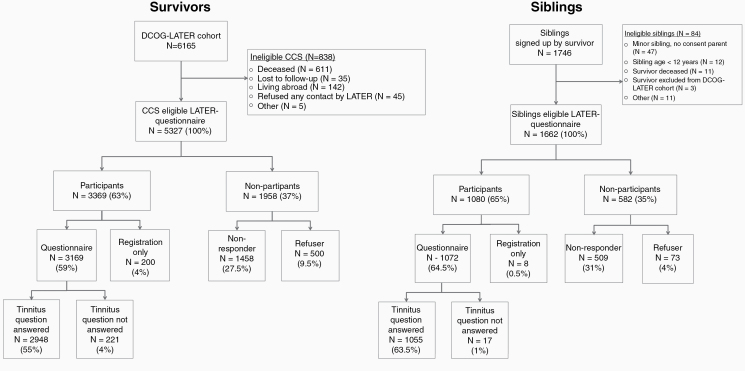
Flowcharts showing the eligibility of CCS and siblings for the general health questionnaire, and representability of those who completed the tinnitus item. CCS, childhood cancer survivors; DCOG, Dutch Childhood Oncology Group.

Among the 6165 survivors, 838 were ineligible for the study. Of the remaining 5327 eligible survivors, 3369 (63%) agreed to participate, and 3169 (59%) returned the general health questionnaire. Among the 1746 siblings, 84 were ineligible for the survey. Of the remaining 1662 siblings, 1080 (65%) agreed to participate in the study, and 1072 (64.5%) returned the general health questionnaire (Figure 1).

The study was approved by the medical ethics boards of all participating centers. Informed consent was obtained from all participants before study inclusion.

### Outcome Measures

The questionnaire included one item on tinnitus, which was the primary outcome of interest. The question was “have you had ringing in the ears, or do you currently have this condition?” (yes/no). A second item ascertained the use of a hearing aid, as follows: “have you had a hearing aid, or do you currently have one?” (yes/no). Survivors and siblings were also asked to fill out the age or year of tinnitus onset and start to wear a hearing aid. The questionnaire did not capture questions on the presence of hearing loss because this was difficult to define for self-reported data.

### Treatment Information

Data on childhood cancer subtype, gender, age at diagnosis, relevant chemotherapy, surgery, and radiotherapy were extracted from the Dutch LATER database on diagnosis and treatment. Detailed information on 45 different types of chemotherapy with total cumulative doses (TCDs) in mg/m^2^ was available. These agents were grouped into 6 categories (ie, alkylating agents, anthracyclines, platinum agents, vinca-alkaloids, antimetabolites, and epipodophyllotoxins). CCS who received radiotherapy were assigned to one of the 2 subgroups: cranial irradiation/total body irradiation (TBI) or irradiation to a body site other than the brain or skull. Data on the total irradiation dose were captured in gray (Gy). Dose reconstructions for structures in the ear were not included as they were not available. Information on surgery was reviewed and divided into 3 categories: surgery on head/cranium (including brain surgery and ventriculostomy), ear, nose, throat (ENT) surgery (including stapedectomy, myringotomy, and tympanoplasty), and surgery to any other body site.

### Data Analysis

Descriptive statistics included medians along with range, and frequencies with percentages were provided for continuous and categorical variables, respectively. Occurrence of self-reported tinnitus (in %) was compared between survivors and siblings. Chi-square tests and Mann–Whitney *U* tests were used to compare clinical characteristics between CCS and siblings.

Generalized estimating equation models were estimated to study the association between tinnitus occurrence in CCS compared to siblings. These models account for the presence of correlation between siblings. The models were adjusted for gender and age at the time of questionnaire. Odds ratios (ORs) with 95% confidence intervals (CIs) were estimated.

Logistic regression models were employed to identify risk factors associated with tinnitus in CCS. Variables with a *P* value of less than .20 in the univariate analyses were included in multivariate analyses. Forward method was applied to estimate the final logistic regression models. All variables associated with tinnitus with a *P* value of less than .05 were included in the final model. ORs along with their 95% CIs were estimated. Gender (male/female), age at diagnosis (<5 years, 5–9 years, 10+ years), cranial irradiation/TBI (yes/no), neuro/ENT surgery (yes/no), and platinum agents (cisplatin and carboplatin, yes/no) were included in one model. The TCDs of platinum agents and the total irradiation doses were analyzed as dichotomous variables in a second model. Cutoff points were based on the literature^9,11,12^ and dose–response analysis (Supplementary Table 1).

Furthermore, we compared CCS with tinnitus who had and had not received ototoxic cancer treatment, and CCS without tinnitus with respect to patient and treatment characteristics. For an in-depth search of risk factors, univariate and multivariate logistic regression models were estimated separately for the 2 largest subpopulations of the DCOG-LATER cohort consisting of acute lymphoblastic leukemia (ALL) survivors and central nervous system (CNS) tumor survivors.

All statistical analyses were performed using SPSS software version 25 (SPSS Inc.).

## Results

### Cohort Characteristics

In total, 2948/3169 (93%) CCS and 1055/1072 (98%) siblings completed the query on tinnitus in the general health questionnaire (Figure 1). Survivors who completed the tinnitus item were representative of the total cohort of eligible CCS concerning the age at diagnosis, gender, platinum agents, neuro/ENT surgery, and cranial irradiation/TBI (Supplementary Table 2).

The median age of CCS at the time of cancer diagnosis was 5.3 years (range 0–18), and the median time from diagnosis to questionnaire completion was 22.4 years (range 11–50; Table 1). Most of the CCS had been diagnosed with ALL (*N* = 888, 30%) and CNS tumors (*N* = 353, 12%). Siblings were more often female (*P* < .001) and older (more often >20 years of age) at time of questionnaire (*P* < .001) compared to CCS.

**Table 1.** Baseline Characteristics of CCS Who Answered the Question on Tinnitus Compared to Siblings, Chi-Square Test Results

	CCS (*N* = 2948)	Siblings (*N* = 1055)	*P*
Childhood cancer type, *n* (%)			
Acute lymphoblastic leukemia	888 (30.0)	NA (NA)	
Acute myeloid leukemia^a^	132 (4.5)	NA (NA)	
Non-Hodgkin lymphoma^b^	329 (11.1)	NA (NA)	
Hodgkin lymphoma	186 (6.3)	NA (NA)	
Central nervous system tumors	353 (12.0)	NA (NA)	
Neuroblastoma	162 (5.5)	NA (NA)	
Retinoblastoma	14 (0.5)	NA (NA)	
Renal tumors	326 (11.1)	NA (NA)	
Hepatic tumors	32 (1.1)	NA (NA)	
Osteosarcoma	86 (2.9)	NA (NA)	
Ewing sarcoma/other bone tumors	81 (2.8)	NA (NA)	
Soft tissue tumors	210 (7.1)	NA (NA)	
Germ cell tumors	108 (3.7)	NA (NA)	
Other and unspecified tumors	41 (1.4)	NA (NA)	
Age at diagnosis in years, *n* (%)			
<5	1379 (46.8)	NA (NA)	
5–9	789 (26.8)	NA (NA)	
10+	780 (26.4)	NA (NA)	
Calendar year childhood cancer diagnosis, *n* (%)			
1963–1984	883 (30.0)	NA (NA)	
1985–1994	1058 (35.9)	NA (NA)	
1995–2001	1007 (34.2)	NA (NA)	
Time since diagnosis to questionnaire in years, *n* (%)			
<20	1207 (40.9)	NA (NA)	
20–29	433 (14.7)	NA (NA)	
30–39	310 (10.5)	NA (NA)	
40+	108 (3.7)	NA (NA)	
Age at questionnaire in years^c^, *n* (%)			<.001
<20	489 (16.6)	115 (11.0)	
20–29	1023 (34.7)	343 (32.8)	
30–39	920 (31.2)	341 (32.6)	
40+	516 (17.5)	248 (23.5)	
Gender, *n* (%)			<.001
Male	1523 (51.7)	446 (42.3)	
Female	1425 (48.3)	609 (57.7)	
Tinnitus, *n* (%)			<.001
No	2668 (90.5)	1015 (96.3)	
Yes	280 (9.5)	40 (3.7)	
Age at start tinnitus in years^d^, *n* (%)			.003
≤30	98 (67.1)	16 (47.1)	
>30	48 (32.9)	18 (52.9)	
Hearing aid use^e^, *n* (%)			<.001
No	2813 (95.6)	1047 (99.3)	
Yes	129 (4.4)	7 (0.7)	
Age at hearing aid use in years^f^, *n* (%)			NA^g^
≤30	39 (78.0)	2 (28.6)	
>30	11 (22.0)	5 (71.4)	

CCS, childhood cancer survivors; NA, not applicable.

^a^Including chronic myeloproliferative diseases, myelodysplastic syndrome, and unspecified/other leukemias.

^b^Including Burkitt lymphomas, miscellaneous lymphoreticular neoplasms, and unspecified lymphomas.

^c^ *N* = 8 missings for siblings.

^d^ *N* = 134 missings for survivors and *N* = 6 missings for siblings.

^e^ *N* = 6 missings for survivors and *N* = 1 for siblings.

^f^ *N* = 79 missings for survivors.

^g^Cannot be executed as the number of siblings ≤30 years of age is lower than 5.

In total, 1968/2948 (67%) survivors underwent surgery, of whom 150 (8%) were carried out on head/cranium or ear (Table 2). Chemotherapy had been administered to 2472/2948 (84%) survivors, of which 374 (15%) received platinum agents. Radiotherapy had been administered to 1147/2948 (39%) CCS. Among these irradiated patients, 698 (61%) received cranial irradiation/TBI.

**Table 2.** Treatment Characteristics of the Total Cohort of CCS, Separated for Those With and Without Tinnitus

	Total CCS Cohort (*N* = 2948)	Tinnitus + (*N* = 280)	Tinnitus− (*N* = 2668)
Surgery, *n* (%)			
No surgery	980 (33.2)	86 (30.7)	894 (33.5)
Neuro/ENT surgery	150 (5.1)	27 (9.6)	123 (4.6)
Other surgery	1818 (61.7)	167 (59.6)	1651 (61.9)
Radiotherapy, *n* (%)			
No irradation	1801 (61.1)	127 (45.5)	1674 (62.7)
Cranial irradiation incl. total body irradiation	698 (23.7)	105 (37.5)	593 (22.2)
Other irradiation site	449 (15.2)	48 (17.1)	401 (15.0)
Chemotherapy, *n* (%)			
No	476 (16.1)	61 (21.8)	415 (15.6)
Yes	2472 (83.9)	219 (78.2)	2253 (84.4)
Alkylating agents, *n* (%)			
No	1528 (51.8)	141 (50.4)	1279 (47.9)
Yes	1420 (48.2)	139 (49.6)	1389 (52.1)
Anthracyclines, *n* (%)			
No	1578 (53.5)	174 (62.1)	1404 (52.6)
Yes	1370 (46.5)	106 (37.9)	1264 (47.4)
Platinum agents, *n* (%)			
No	2574 (87.3)	233 (83.2)	2341 (87.7)
Yes	374 (12.7)	47 (16.8)	327 (12.3)
Vinca-alkaloids, *n* (%)			
No	740 (25.1)	98 (35.0)	642 (24.1)
Yes	2208 (74.9)	182 (65.0)	2026 (75.9)
Antimetabolites, *n* (%)			
No	1549 (52.5)	156 (55.7)	1393 (52.2)
Yes	1399 (47.5)	124 (44.3)	1275 (47.8)
Epipodophyllotoxins, *n* (%)			
No	2363 (80.2)	233 (83.2)	2130 (79.8)
Yes	585 (19.8)	47 (16.8)	538 (20.2)

CCS, childhood cancer survivors; ENT, ear, nose, and throat.

### Occurrence of Tinnitus in CCS Compared to Siblings

Tinnitus occurred in 280/2948 (9.5%) CCS compared to 40/1055 (3.7%) in siblings (Table 1). Age at tinnitus onset was available for 146/280 (52%) survivors and 34/40 (85%) siblings. Median age of tinnitus onset in CCS was 25 years (range 4–54) and in siblings 35 years (range 5–53). Among these survivors, the median time from diagnosis to tinnitus onset was 15 years (range 0–39). The OR for tinnitus occurrence in CCS compared to siblings was 3.0 (95% CI 2.9–3.1), adjusted for gender and age at the questionnaire. In total, 82/280 (29%) of the CCS with tinnitus wore a hearing aid (3% of the total CCS cohort) compared to 2/40 (5%) siblings (0.2% of the total siblings cohort). Tinnitus most often occurred in CCS who had been diagnosed with osteosarcoma (18/86% = 21%), CNS tumors (57/353 = 16%), Ewing sarcoma (11/81 = 14%), and other tumors (7/41 = 17%; Supplementary Table 3).

### Risk Factors Associated With Tinnitus in CCS

In Table 3, estimated ORs from univariate and multivariate logistic regression models are provided. The univariate analysis showed that tinnitus occurrence in CCS was associated with age at diagnosis, neuro/ENT surgery, (total dose) cranial irradiation/TBI, and (TCD) cisplatin, but not with gender and (TCD) carboplatin.

**Table 3.** Risk Factors Associated With Tinnitus Comparing CCS With and Without Tinnitus, Univariate and Multivariate Results

	Tinnitus +	Tinnitus−	UVA	MVA^a^	MVA^b^
	*N* = 280 (%)	*N* = 2668 (%)	OR (95% CI)	OR (95% CI)	OR (95% CI)
Gender					
Male	151 (54)	1372 (51)	REF		
Female	129 (46)	1296 (49)	0.9 (0.7–1.2)	-	-
Age at diagnosis					
<5 years	96 (34)	1283 (48)	REF	REF	REF
5–9 years	72 (26)	717 (27)	1.3 (1.0–1.8)*	1.2 (0.9–1.7)	1.2 (0.9–1.7)
10+ years	112 (40)	668 (25)	2.2 (1.7–3.0)*	2.1 (1.5–2.8)**	2.1 (1.6–2.8)**
Neuro/ENT surgery					
No	253 (90)	2545 (95)	REF	REF	REF
Yes	27 (10)	123 (5)	2.2 (1.4–3.4)*	1.8 (1.1–2.8)**	1.8 (1.1–2.9)**
Cranial irradiation/TBI					
No	175 (63)	2075 (78)	REF	REF	
Yes	105 (37)	593 (22)	2.1 (1.6–2.7)*	1.9 (1.5–2.5)**	-
TD cranial irradiation/TBI					
No irradiation	175 (63)	2075 (78)	REF		REF
<50 Gy	62 (22)	391 (15)	1.9 (1.4–2.6)*	-	1.9 (1.4–2.7)**
≥50 Gy	42 (15)	193 (7)	2.6 (1.8–3.7)*		1.9 (1.3–2.9)**
TD cranial irradiation/TBI					
<50 Gy	62 (60)	391 (67)	REF		
≥50 Gy	42 (40)	193 (33)	1.4 (0.9–2.1)	-	-
Carboplatin					
No	263 (94)	2487 (93)	REF		
Yes	17 (6)	181 (7)	0.9 (0.5–1.5)	-	-
TCD carboplatin					
No carboplatin	263 (94)	2.487 (93)	REF		
<1500 mg/m^2^	5 (2)	50 (2)	0.9 (0.4–2.4)	-	-
≥1500 mg/m^2^	11 (4)	125 (5)	0.8 (0.4–1.6)		
TCD carboplatin					
<1500 mg/m^2^	5 (31)	50 (29)	REF	-	-
≥1500 mg/m^2^	11 (69)	125 (71)	0.9 (0.3–2.7)		
Cisplatin					
No	243 (87)	2499 (94)	REF	REF	
Yes	37 (13)	169 (6)	2.3 (1.5–3.3)*	2.0 (1.4–3.0)**	-
TCD cisplatin					
No cisplatin	243 (87)	2499 (94)	REF		REF
<400 mg/m^2^	15 (5)	69 (3)	2.2 (1.3–4.0)*	-	1.8 (0.9–3.2)
≥400 mg/m^2^	21 (8)	91 (3)	2.3 (1.5–3.9)*		2.4 (1.4–4.0)**
TCD cisplatin					
<400 mg/m^2^	15 (42)	69 (43)	REF		
≥400 mg/m^2^	21 (58)	91 (57)	1.1 (0.5–2.2)	-	-

CCS, childhood cancer survivors; CI, confidence interval; MVA, multivariate analysis; OR, odds ratio; REF, reference; TBI, total body irradiation; TD, total dose; TCD, total cumulative dose; UVA, univariate analysis.

^a^Model with cranial irradiation and cisplatin.

^b^Model with cranial irradiation dose and cisplatin dose (patients without treatment as reference group).

*P < .2, **P < .05.

Two multivariate logistic regression models were estimated. Model 1 included cranial irradiation/TBI and cisplatin treatment as dichotomous variables (yes/no); in model 2, total dose of cranial irradiation (<50 Gy and ≥50 Gy) and TCD of cisplatin (<400 mg/m^2^ and ≥400 mg/m^2^) were included using CCS without these treatments as the reference category.

Multivariate model 1 revealed that tinnitus occurrence was significantly associated with age at diagnosis (≥10 years: OR 2.1, 95% CI 1.5–2.8), cisplatin treatment (OR 2.0, 95% CI 1.4–3.0), cranial irradiation/TBI (OR 1.9, 95% CI 1.5–2.5), and neuro/ENT surgery (OR 1.8, 95% CI 1.1–2.8). Multivariate model 2 identified TCD cisplatin ≥400 mg/m^2^ (OR 2.4, 95% CI 1.4–4.0), age at diagnosis (≥10 years: OR 2.1, 95% CI 1.6–2.8), and neuro/ENT surgery (OR 1.8, 95% CI 1.1–2.9) as independent factors for tinnitus occurrence. The ORs for tinnitus occurrence were the same (OR 1.9) for the 2 subgroups according to cranial irradiation dose (<50 Gy/≥50 Gy).

### Additional Risk Factors for Tinnitus in CCS

A total of 142/280 (51%) CCS with tinnitus had received either cisplatin, cranial irradiation/TBI, and/or neuro/ENT surgery, whereas 138/280 (49%) had not (Supplementary Figure 1). The latter group of CCS had frequently been treated between 1985 and 1994, were often aged 5 years or younger at the time of diagnosis and 30 years or younger at the time of questionnaire. Furthermore, they wore a hearing aid (50/138, 36%) more often than CCS who had received cisplatin, cranial irradiation/TBI, or neuro/ENT surgery (32/142, 23%), and CCS without tinnitus (47/2668, 2%; Supplementary Table 3).

In the subpopulation of ALL survivors, tinnitus occurred in 72/888 (8%) CCS (Supplementary Figure 2) compared to 9/320 (3%) siblings (OR 3.3, 95% CI 3.2–3.4), adjusted for gender and age at questionnaire. A total of 281/888 (32%) ALL survivors received cranial irradiation/TBI, whereas 607/888 (68%) had not. Among them, 32/72 (44%) CCS with tinnitus had received cranial irradiation/TBI (Supplementary Figure 1). Univariate analysis showed that ALL survivors had a higher risk of tinnitus when they were treated before 1985 (OR 2.2, 95% CI 1.2–4.0), were 10 years or older at the time of diagnosis (OR 2.1, 95% CI 1.2–3.8), and/or had received cranial irradiation/TBI (OR 1.8, 95% CI 1.1–3.0). According to the multivariate analysis, age at diagnosis and cranial irradiation/TBI were significantly associated with tinnitus occurrence in ALL survivors (OR 2.1, 95% CI 1.1–3.8; OR 1.8, 95% CI 1.1–3.0, respectively).

Among survivors who had been diagnosed with CNS tumors, tinnitus occurred in 57/353 (16%) CCS (Supplementary Figure 2), compared to 1/111 (0.9%) siblings. Most of the CCS with tinnitus had been diagnosed with medulloblastoma (*N* = 22, 39%), low-grade astrocytoma (*N* = 13, 23%), and non-low-grade astrocytoma (*N* = 10, 18%). In total, 210/353 (59%) CCS had received cranial irradiation (39/57 [68%]: with tinnitus), 34/353 (10%) CCS had received cisplatin (8/57 [14%]: with tinnitus), 68/353 (19%) CCS had received carboplatin (8/57 [14%]: with tinnitus), and 108/353 (31%) had received neuro/ENT surgery (21/57 [37%]: with tinnitus). Univariate analysis showed that CNS tumor survivors had a higher risk of tinnitus when they were treated before 1985 (OR 2.9, 95% CI 1.3–6.3), were of male gender (OR 1.5, 95% CI 0.8–2.6), were 10 years or older at time of diagnosis (OR 1.8, 95% CI 0.9–3.8), and/or had received cranial irradiation (OR 1.6, 95% CI 0.9–2.9). In the multivariate analyses, only the treatment period remained significantly associated with tinnitus occurrence in CNS tumor survivors (before 1985: OR 2.6, 95% CI 1.2–5.9).

## Discussion

This study explored the occurrence of and risk factors for tinnitus in a national cohort of CCS. Our results revealed that tinnitus occurred in 9.5% of CCS, and they were nearly 3 times more likely to suffer from tinnitus compared to siblings. Age at diagnosis, neuro/ENT surgery, cranial irradiation/TBI, and cisplatin (TCD ≥400 mg/m^2^) were identified as independent risk factors. The latter treatment modalities had been administered to about 50% of the CCS who reported tinnitus, which suggests that also other factors might be associated with the occurrence of tinnitus in this population.

To date, many studies investigating audiological acute and late effects of childhood cancer treatment have focused on hearing loss. However, information on the frequency of and risk factors for tinnitus during and after childhood cancer based on national cohort studies is limited. In the few available studies, tinnitus was reported to occur in 3–60% of CCS.^11,13–21^ Variation in study sample size, type of childhood cancer, age at diagnosis, cancer treatment, and time to follow-up may have influenced these results. Our recently published systematic review^22^ included a risk of bias assessment by use of the QUIPS tool^23^ among the available studies on tinnitus during and after childhood cancer, which revealed a smaller range of tinnitus in studies of adequate quality (3–17%).^11,13–17^ However, evidence for risk factors of tinnitus within these studies was scarce, indicating that more research was needed. For that purpose, the current study was entirely focused on identifying tinnitus and its risk factors, and it was shown that the estimated proportion of 9.5% in CCS is higher than in controls.

The results of this study indicate that CCS had more odds of developing tinnitus compared to a sibling control group that was relatively older. This finding is important as tinnitus can have a large negative impact on quality of life. Adults with bothersome ringing in the ears often have to cope with accompanying symptoms such as concentration problems and insomnia, which are known to frequently lead to depression, anxiety, and mental distress. Consequently, work performance and social participation can be affected.^24^ Clinical management strategies that can be helpful for a subset of tinnitus patients are available. This includes mainly psychological treatment such as cognitive behavioral therapy and/or auditory stimulation by the use of hearing aids or personal listening devices.^25^ Auditory stimulation restores or enhances afferent input to the auditory neural pathways by providing or amplifying (environmental) sounds, leading to downregulation of spontaneous neural activity, and thereby reducing the comparative loudness of tinnitus in quiet environments.^26^ Regular surveillance may be helpful for increased awareness, (early) detection, referral, and treatment of tinnitus in CCS.

A high TCD cisplatin was associated with tinnitus occurrence in CCS. This finding is in agreement with the results of previous studies with smaller sample sizes. Cisplatin harms the outer hair cells (OHCs) in the cochlea by damaging DNA and increasing the release of reactive oxygen species,^27^ especially when the drug is administered in high TCDs of 400 mg/m^2^ or more.^12^ Carboplatin did not appear to be associated with tinnitus in the current study. This platinum agent has ototoxic potential but generally causes less cochlear damage compared to cisplatin,^28^ which may explain the low frequency of tinnitus in CCS treated with carboplatin. Furthermore, previously administered cranial irradiation/TBI was associated with the occurrence of tinnitus, but not the total radiation dose. Radiotherapy leads to changes in cochlear OHCs and vascular degeneration and decreases the function of the basilar membrane and cochlear nerve.^29^ To date, conflicting results have been reported regarding the effect of total radiation dose on tinnitus presence.^11,13,15^ It will be important to include dosimetry in future studies to determine the specific radiation dosages on the cochlea and possibly other relevant structures such as the VIII cranial nerve for a better risk estimation of tinnitus development. Lastly, neuro/ENT surgery was identified as a risk factor for tinnitus in CCS. Previous smaller studies have shown that cochlear or neural damage might be related to surgical resection of a CNS tumor and insertion of a cerebrospinal fluid shunt.^4^ As high TCDs cisplatin, cranial irradiation, and neuro/ENT surgery are used in various childhood cancer treatment protocols, increased awareness for tinnitus development in pediatric oncology patients and survivors seems warranted.

About half of CCS with tinnitus had not received cisplatin, cranial irradiation/TBI, and/or neuro/ENT surgery, suggesting that other factors might be associated with tinnitus in this subpopulation. Among CCS, the median time from cancer diagnosis to questionnaire completion was comparatively long (22 years). During this time, several other conditions underlying tinnitus symptoms, which commonly occur in the general population, could have been developed. These include otologic disorders (eg, otosclerosis, Menière’s disease), neurological conditions (eg, head injury, whiplash, multiple sclerosis), infectious diseases (eg, otitis media, Lyme disease, meningitis), and temporomandibular joint dysfunction as well as other dental disorders.^30^ Furthermore, increasing age of CCS could have been associated with tinnitus development. The aging process causes changes in adaptive and compensatory neural mechanisms in the brain, and the presence of tinnitus and its perception might depend upon these mechanisms. It seems that the efficiency of this compensatory mechanism decreases with age, making older individuals more aware of tinnitus.^31^ Lastly, frequent exposure to excessive noise (levels of 90 dB or higher) can induce tinnitus by progressive cochlear hair cell destruction. Noise exposure can be related to work (eg, loud machinery), music at hazardous volume (eg, at concerts, night clubs, or from personal listening devices), or trauma (eg, a sudden loud bang or explosion).^32^ The physiological risk of tinnitus in the general population might be represented in the subgroup of CCS with tinnitus but without treatment-related risk factors. It should be mentioned that siblings in this study were relatively older at the time of questionnaire compared to CCS, indicating that they also might have been exposed to one or more factors mentioned above. Nevertheless, the proportion of tinnitus in siblings remains lower compared to CCS.

In the largest diagnostic subcohort of ALL survivors, 8% suffered from tinnitus, and of them, 56% had not been irradiated. Up to 1985, all pediatric leukemia patients in the Netherlands received cranial irradiation, whereafter it was only applied in high-risk patients with CNS involvement.^33^ Another possible risk factor for tinnitus in these ALL survivors could be the administration of intrathecal therapy, which has substituted cranial irradiation in a more recent treatment era. It is known that this CNS-directed therapy increases the risk of neurologic toxicities,^34^ and a small study reported an association between intrathecal methotrexate and auditory pathway impairment in the brainstem.^35^ Furthermore, fluid overload and infections are important complications during ALL treatment, for which respectively diuretics and antibiotics are often applied. Many of these agents are potentially ototoxic, including aminoglycosides (eg, gentamicin, tobramycin), loop diuretics (eg, furosemide), and glycopeptides (eg, vancomycin).^27^ The specific role of these agents to be associated with cochlear damage without concomitant administration of platinum agents and cranial irradiation needs to be determined. The Dutch LATER study was not designed to capture co-medication use during childhood cancer treatment. Prospective studies are necessary to investigate the role of intrathecal therapy and co-medication on tinnitus development in children with cancer.

To the best of our knowledge, this is the first study in CCS that was entirely dedicated to tinnitus, based on a large national cohort. The inclusion of siblings as a healthy control group was beneficial as CCS and siblings broadly have the same genetic and socio-demographic profile.^36^ Strong conclusions on the reason for and benefit of hearing aid use cannot be drawn from this study (Supplementary Table 4), as the Dutch LATER study was neither designed to capture data on nor to study hearing loss. In addition, as tinnitus can only be heard by an affected individual, it can only be reported in a subjective manner. In the current study, the general health questionnaire was designed to gain insight into the presence of tinnitus in CCS but not on its severity or laterality, leaving it unsure how many mild, moderate, or severe tinnitus cases were identified, and whether there were more unilateral or bilateral tinnitus cases in our cohort. Therefore, it would be useful to include validated tinnitus questionnaires as part of standard toxicity screening in CCS in order to scale the severity and negative impact of tinnitus. The question on age or year of tinnitus onset could have caused recall bias, as it might have been difficult for CCS to remember when tinnitus had developed over time. Potential selection bias may have occurred as 63% of the CCS who were eligible for the questionnaire participated in the study, which might have limited the generalizability of the results. However, we found no statistical differences between CCS who completed the item on tinnitus and CCS who did not.

In conclusion, this study reveals that tinnitus more often occurs in CCS compared to healthy siblings. Awareness of tinnitus in survivors who have been treated with cisplatin, cranial irradiation/TBI, and/or neuro/ENT surgery is warranted among health care professionals. However, these treatment modalities only explain the presence of tinnitus in about half of the CCS. This suggests that other (co-treatment) factors may be involved in tinnitus development in this population, which should be further investigated in future (late effects) studies. Our data show that tinnitus as part of ototoxicity screening during and after childhood cancer deserves serious attention.

## Supplementary Material

vdaa122_suppl_Supplementary_Figures-S1-S2_Tables_S1-S4
