# Supplementary material for: Risk factors associated with tinnitus in 2948 Dutch survivors of childhood cancer: a Dutch LATER questionnaire study
Source: Neurooncol Adv. 2020 Sep 15;2(1):vdaa122. doi: 10.1093/noajnl/vdaa122 (PMC7648591; doi:10.1093/noajnl/vdaa122)
Supplement: vdaa122_suppl_Supplementary_Figures-S1-S2_Tables_S1-S4 [file vdaa122_suppl_supplementary_figures-s1-s2_tables_s1-s4.docx]

| **Supp. Table 1. Cut-off points of cisplatin, carboplatin and cranial irradiation/TBI doses comparing CCS with and without tinnitus, univariate and multivariate results.** | | | | |
| --- | --- | --- | --- | --- |
|  | **Tinnitus +**  **N=280 (%)** | **Tinnitus -**  **N=2,668 (%)** | **UVA**  **OR (95% CI)** | **MVA^a^**  **OR (95% CI)** |
| TCD cisplatin per 100mg/m^2^ increase | 400 (75-990) | 400 (80-720) | 0.9 (0.8-1.0) | - |
| No cisplatin | 243 (87) | 2,499 (94) | REF | REF |
| First quartile (0-300 mg/m^2^) | 6 (2) | 36 (1) | 1.7 (0.7-4.1) | 1.6 (0.7-3.9) |
| Second quartile (300-400 mg/m^2^) | 9 (3) | 33 (1) | 2.8 (1.3-5.9)* | 2.4 (1.1-5.2)** |
| Third quartile (400-570 mg/m^2^) | 14 (5) | 49 (2) | 2.9 (1.6-5.4)* | 2.9 (1.6-5.3)** |
| Fourth quartile (≥570 mg/m^2^) | 7 (3) | 42 (2) | 1.7 (0.8-3.8) | 1.5 (0.7-3.4) |
| TCD carboplatin per 500mg/m^2^ increase | 2100 (600-7200) | 2200 (158-17,920) | 1.0 (1.0-1.0) | - |
| No carboplatin | 263 (94) | 2,487 (93) |  |  |
| First quartile (0-1,500 mg/m^2^) | 4 (1.5) | 36 (1) |  |  |
| Second quartile (1,500-2,200 mg/m^2^) | 4 (1.5) | 50 (2) | NA^#^ | NA^#^ |
| Third quartile (2,200-3,360 mg/m^2^) | 2 (1) | 47 (2) |  |  |
| Fourth quartile (≥3,360 mg/m^2^) | 6 (2) | 42 (2) |  |  |
| TD cranial irradiation/TBI per 5 Gy increase | 39 (2-96) | 25 (2-146) | 1.0 (0.9-1.1)* | 1.0 (0.9-1.1) |
| No irradiation | 175 (63) | 2,075 (78) | REF | REF |
| First quartile (0-22 Gy) | 21 (8) | 151 (6) | 1.6 (1.0-2.7)* | 1.6 (1.0-2.6) |
| Second quartile (22-25 Gy) | 7 (2) | 42 (2) | 1.9 (0.9-4.5)* | 2.0 (0.9-4.6) |
| Third quartile (25-50 Gy) | 34 (12) | 198 (7) | 2.0 (1.4-3.0)* | 2.1 (1.4-3.1)** |
| Fourth quartile (≥50 Gy) | 42 (15) | 193 (7) | 2.6 (1.8-3.7)* | 2.3 (1.6-3.4)** |
| * p <0.2; ** p < 0.05. ^a^ Model adjusted for age at diagnosis. ^#^ NA: not applicable to calculate ORs due n=5. *Abbreviations:* CI = confidence interval; MVA = multivariate analysis; OR = odds ratio; REF = reference; TBI = total body irradiation; TD = total dose; TCD = total cumulative dose; UVA = univariate analysis. | | | | |

| **Supp. Table 2.** **Patient and treatment characteristics of CCS who completed the tinnitus item in the questionnaire compared to those who did not, Chi-square test results.** | | | | | | |
| --- | --- | --- | --- | --- | --- | --- |
|  | **CCS who did answer  tinnitus question** | | | **CCS who did not  answer tinnitus  question** | |  |
|  | **(N=2,948)** | | | **(N=221)** | | ***P*** |
| Age at diagnosis in years, n (%) |  | | |  | | 0.9 |
| <5 | 1,379 | | (47) | 106 | (48) |  |
| 5-9 | 789 | | (27) | 59 | (27) |  |
| 10+ | 780 | | (26) | 56 | (25) |  |
| Gender, n (%) |  | | |  | | 0.2 |
| Male | 1,523 | (52) | | 125 | (57) |  |
| Female | 1,425 | (48) | | 96 | (43) |  |
| Platinum agent, n (%) |  | | |  | | 0.6 |
| No | 2,574 | (87) | | 190 | (86) |  |
| Yes | 374 | (13) | | 31 | (14) |  |
| Cranial irradiation/TBI, n (%) |  | | |  | | 0.8 |
| No | 2,250 | (76) | | 167 | (76) |  |
| Yes | 698 | (24) | | 54 | (24) |  |
| Neuro/ENT surgery, n (%) |  | | |  | | 0.9 |
| No | 2,798 | (95) | | 210 | (95) |  |
| Yes | 150 | (5) | | 11 | (5) |  |
| *Abbreviations:* CCS = childhood cancer survivors; ENT = ear, nose and throat; TBI = total body irradiation. | | | | | | |

| **Supp. Table 3. Patient and treatment characteristics of CCS with tinnitus comparing those who had and had not received ototoxic cancer treatment, and CCS without tinnitus.** | | | | | | |
| --- | --- | --- | --- | --- | --- | --- |
|  | **CCS with tinnitus, without CRT/TBI, cisplatin, and neuro/ENT surgery** | | **CCS with tinnitus, with CRT/TBI, cisplatin, or neuro/ENT surgery** | | **CCS without  tinnitus** | |
|  | **(N=138)** | | **(N=142)** | | **(N=2,668)** | |
| Gender, n (%) |  |  |  |  |  |  |
| Male | 74 | (54) | 77 | (54) | 1,372 | (51) |
| Female | 64 | (46) | 65 | (46) | 1,296 | (49) |
| Age at diagnosis in years, n (%) |  |  |  |  |  |  |
| <5 | 56 | (41) | 40 | (28) | 1,283 | (48) |
| 5-9 | 36 | (26) | 36 | (25) | 717 | (27) |
| 10+ | 46 | (33) | 66 | (47) | 668 | (25) |
| Calendar year childhood cancer diagnosis, n (%) |  |  |  |  |  |  |
| 1963-1984 | 42 | (30) | 66 | (46) | 775 | (29) |
| 1985-1994 | 60 | (44) | 51 | (36) | 947 | (36) |
| 1995-2001 | 36 | (26) | 25 | (18) | 946 | (35) |
| Age at questionnaire in years, n (%) |  |  |  |  |  |  |
| <20 | 16 | (12) | 4 | (3) | 469 | (18) |
| 20-29 | 56 | (41) | 29 | (20) | 938 | (35) |
| 30-39 | 34 | (25) | 54 | (38) | 832 | (31) |
| 40+ | 32 | (23) | 55 | (39) | 429 | (16) |
| Time since diagnosis in years, n (%) |  |  |  |  |  |  |
| <20 | 45 | (33) | 33 | (23) | 1,129 | (42) |
| 20-29 | 56 | (41) | 53 | (37) | 887 | (33) |
| 30-39 | 26 | (19) | 49 | (35) | 562 | (21) |
| 40+ | 11 | (8) | 7 | (5) | 90 | (4) |
| Childhood cancer diagnosis, n (%) |  |  |  |  |  |  |
| Acute lymphoblastic leukemia | 40 | (29) | 32 | (22) | 816 | (31) |
| Acute myeloid leukemia | 1 | (1) | 11 | (8) | 120 | (4) |
| Non-Hodgkin lymphoma | 16 | (11) | 6 | (4) | 307 | (11) |
| Hodgkin lymphoma | 12 | (9) | 4 | (3) | 170 | (6) |
| Central nervous system tumors | 8 | (6) | 49 | (35) | 296 | (11) |
| Neuroblastoma | 5 | (4) | 5 | (3) | 152 | (6) |
| Retinoblastoma | 1 | (1) | 0 | (0) | 13 | (1) |
| Renal tumors | 22 | (16) | 0 | (0) | 304 | (11) |
| Hepatic tumors | 0 | (0) | 1 | (1) | 31 | (1) |
| Osteosarcoma | 4 | (3) | 14 | (10) | 68 | (3) |
| Ewing sarcoma/other bone tumors | 10 | (7) | 1 | (1) | 70 | (3) |
| Soft tissue tumors | 13 | (9) | 6 | (4) | 191 | (7) |
| Germ cell tumors | 3 | (2) | 9 | (6) | 96 | (4) |
| Other and unspecified tumors | 3 | (2) | 4 | (3) | 34 | (1) |
| Hearing aid use, n (%) |  |  |  |  |  |  |
| No | 88 | (64) | 110 | (77) | 2,621 | (98) |
| Yes | 50 | (36) | 32 | (23) | 47 | (2) |
| *Abbreviations:*  CCS = childhood cancer survivors; CRT = cranial irradiation; ENT = ear, nose and throat; TBI = total body irradiation. | | | | | | |

| **Supp. Table 4. Risk factors associated with hearing aid use comparing CCS with and without a hearing aid, univariate and multivariate results.** | | | | | | |
| --- | --- | --- | --- | --- | --- | --- |
|  | **Hearing aid +** | **Hearing aid -** | **UVA** | **MVA^a^** | **MVA^b^** | **MVA^c^** |
|  | **N=129 (%)** | **N=2,813 (%)** | **OR (95% CI)** | **OR (95% CI)** | **OR (95% CI)** | **OR (95% CI)** |
| **Gender**  Male  Female | 70 (54)  59 (46) | 1,450 (52)  1,363 (48) | REF  0.9 (0.6-1.3) | - | - | - |
| **Age at diagnosis**  <5 years  5-9 years  10+ years | 57 (44)  37 (29)  35 (27) | 1,319 (47)  749 (27)  745 (26) | REF  1.1 (0.7-1.8)  1.1 (0.7-1.7) | - | - | - |
| **Neuro/ENT surgery**  No  Yes | 110 (85)  19 (15) | 2,682 (95)  131 (5) | REF  3.5 (2.1-5.9)* | REF  2.6 (1.5-4.5)** | REF  2.0 (1.1-3.6)** | REF  1.7 (0.8-3.6) |
| **Cranial irradiation/TBI**  No  Yes | 80 (62)  49 (38) | 2,164 (77)  649 (23) | REF  2.0 (1.4-2.9)* | REF  1.9 (1.3-2.7)** | - | - |
| **TD cranial irradiation/TBI**  No irradiation  <50 Gy  ≥50 Gy | 80 (63)  16 (12)  32 (25) | 2,164 (77)  437 (16)  203 (7) | REF  1.0 (0.6-1.7)  4.2 (2,8-6.6)* | - | REF  1.1 (0.6-1.9)  3.4 (2.1-5.4)** | - |
| **TD cranial irradiation/TBI**  <50 Gy  ≥50 Gy | 16 (33)  32 (67) | 437 (68)  203 (32) | REF  4.3 (2.3-8.0)* | - | - | REF  3.1 (1.6-6.1)** |
| **Carboplatin**  No  Yes | 119 (92)  10 (8) | 2,625 (93)  188 (7) | REF  1.2 (0.6-2.3) | - | - | - |
| **TCD carboplatin**  No carboplatin  <1500 mg/m^2^  ≥1500 mg/m^2^ | 119 (92)  2 (2)  8 (6) | 2,625 (94)  53 (2)  128 (5) | NA^#^ | NA^#^ | NA^#^ | NA^#^ |
| **TCD carboplatin**  <1500 mg/m^2^  ≥1500 mg/m^2^ | 2 (20)  8 (80) | 53 (29)  128 (71) | NA^#^ | NA^#^ | NA^#^ | NA^#^ |
| **Cisplatin**  No  Yes | 106 (82)  23 (18) | 2,630 (94)  183 (6) | REF  3.1 (1.9-5.0)* | REF  2.9 (1.8-4.7)** | - | REF  2.9 (1.3-6.5)** |
| **TCD cisplatin**  No cisplatin  <400 mg/m^2^  ≥400 mg/m^2^ | 106 (82)  8 (6)  15 (12) | 2,630 (94)  76 (3)  97 (3) | REF  2.6 (1.3-5.6)*  3.8 (2.1-6.8)* | - | REF  1.7 (0.8-3.8)  3.7 (2.0-6.7)** | - |
| **TCD cisplatin**  <400 mg/m^2^  ≥400 mg/m^2^ | 8 (35)  15 (65) | 76 (44)  97 (56) | REF  1.5 (0.6-3.6) | - | - | - |
| * p <0.2; ** p < 0.05. ^a^ Model with cranial irradiation and cisplatin; ^b^ Model with cisplatin dose and cranial irradiation dose (patients without treatment as reference group).  ^c^ Model with cranial irradiation dose (patients without lower dose as reference group). ^#^NA: not applicable to calculate ORs due n=5. *Abbreviations:* CCS = childhood cancer survivors; CI = confidence interval; MVA = multivariate analysis; OR = odds ratio; REF = reference; TBI = total body irradiation; TD = total dose; TCD = total cumulative dose; UVA = univariate analysis. | | | | | | |

**N=12**

**N=3**

**N=5**

**N=13**

**Supp. Figure 1. Overlap in ototoxic treatments for childhood cancer among the total cohort of CCS with tinnitus (N=280), and for ALL survivors with tinnitus (N=72); see also supplementary table 3 for a subset description of CCS with tinnitus who did not receive cisplatin, cranial irradiation, or neuro/ENT surgery.**

*Abbreviations*: ALL = acute lymphoblastic leukemia; ENT = ear, nose, and throat; TBI = total body irradiation.

**Supp. Figure 2. Distribution with respect to diagnosis subtypes of CCS with tinnitus (N=280).**

*Abbreviations*: ALL = acute lymphoblastic leukemia; AML = acute myeloid leukemia; CNST = central nervous system tumor; ES/other = Ewing sarcoma/other bone tumors; GCT = germ cell tumor; HL = Hodgkin lymphoma; HT = hepatic tumors; NBL = neuroblastoma; NHL = non-Hodgkin lymphoma; OS = osteosarcoma; Other/unsp. = other and unspecified tumors; RBL = retinoblastoma; RT = renal tumors; STS = soft tissue sarcoma.
